# Mass Stranding of Marine Birds Caused by a Surfactant-Producing Red Tide

**DOI:** 10.1371/journal.pone.0004550

**Published:** 2009-02-23

**Authors:** David A. Jessup, Melissa A. Miller, John P. Ryan, Hannah M. Nevins, Heather A. Kerkering, Abdou Mekebri, David B. Crane, Tyler A. Johnson, Raphael M. Kudela

**Affiliations:** 1 California Department of Fish and Game, Marine Wildlife Veterinary Care and Research Center (MWVCRC), Santa Cruz, California, United States of America; 2 Monterey Bay Aquarium Research Institute, Moss Landing, California, United States of America; 3 Moss Landing Marine Laboratories, Moss Landing, California, United States of America; 4 CeNCOOS Program, Monterey Bay Aquarium Research Institute, Moss Landing, California, United States of America; 5 California Department of Fish and Game, Water Pollution Control Laboratory, Rancho Cordova, California, United States of America; 6 Ocean Sciences Department and Institute for Marine Sciences, University of California Santa Cruz, Santa Cruz, California, United States of America; University of Kent, United Kingdom

## Abstract

In November-December 2007 a widespread seabird mortality event occurred in Monterey Bay, California, USA, coincident with a massive red tide caused by the dinoflagellate *Akashiwo sanguinea*. Affected birds had a slimy yellow-green material on their feathers, which were saturated with water, and they were severely hypothermic. We determined that foam containing surfactant-like proteins, derived from organic matter of the red tide, coated their feathers and neutralized natural water repellency and insulation. No evidence of exposure to petroleum or other oils or biotoxins were found. This is the first documented case of its kind, but previous similar events may have gone undetected. The frequency and amplitude of red tides have increased in Monterey Bay since 2004, suggesting that impacts on wintering marine birds may continue or increase.

## Introduction

Harmful algal blooms (HABs) caused by a variety of dinoflagellates occur worldwide and can cause morbidity and mortality of fish, invertebrates, marine birds, marine mammals and humans [1]–[4]. The most prevalent form of morbidity and mortality in marine organisms results from direct ingestion, inhalation, or absorption of toxins, including the potent neurotoxins domoic acid, saxitoxin, and brevetoxin [5]–[8]. Particularly notable events include domoic acid poisoning of California sea lions, common dolphins, southern sea otters, and whales [7], [9]–[11] caused by ingestion of algae or contaminated prey; brevetoxin poisoning of manatees caused by direct inhalation [12] and poisoning of manatees and dolphins by ingestion of contaminated fish and seagrasses [13]; paralytic shellfish poisoning of right whales caused by ingestion of contaminated copepods [14]. Morbidity and mortality, particularly for fish, can also occur through direct physical contact with some HAB organisms (e.g. *Pfiesteria* spp., [15]) or from hypoxia/anoxia that develops during bloom respiration and senescence [2].

HAB events have been linked to mortality of marine birds, especially piscivorous species, since the late Pliocene (see reviews by [3], [4], [9]). Exposure to marine toxins has resulted in acute stranding and mortality events [2], [3], [8], as well as in documented behavioral changes to minimize toxin exposure [16]. Herein we present the first report of harmful effects caused by external coating of marine birds by a proteinaceous foam derived from a red tide bloom that occurred November 2007 in Monterey Bay, CA (USA). Although this red tide bloom was ostensibly nontoxic, it was very harmful, causing unprecedented beach stranding of live and dead seabirds. This is the first documented case of its kind, but previous similar events worldwide may have gone undetected.

Monterey Bay is an important foraging and molting area for a diversity of migratory birds, second only to San Francisco Bay in importance to sea ducks (*Melanitta* spp.) and nearshore species wintering along the west coast of the United States. The species affected in this HAB event also are among those typically affected by winter oil spills in the region, including loons (*Gavia* spp.) and grebes (*Aechmophorus* spp.) [17]. Sensitive species also were affected such as red-throated loons (*G. stellata*) which have shown significant breeding population declines in recent years. Being entirely aquatic, loons and grebes do not come on land during winter and are dependent upon maintaining waterproof plumage to maintain their thermal balance and foraging activities. Similar to the impacts of an oiling event, a plumage-fouling agent of any kind can compromise waterproofing and essentially disable an otherwise healthy individual.

## Results

Fourteen seabird species were affected during the 2007 red tide in Monterey Bay, particularly those that feed in near shore habitat, such as surf scoters (*Melanitta perspicillata*), Pacific and red-throated loons (*Gavia* spp.), and Clark's and western grebes (*Aechmorphorus* spp.). Some of these, including the loons, grebes and northern fulmar (*Fulmarus glacialis*), had just completed their southward migration and were in poor nutritional condition prior to this event. A total of 550 stranded alive and 207 were collected fresh dead during this event. The majority of birds were fulmars (n = 245).

**Figure 1:**
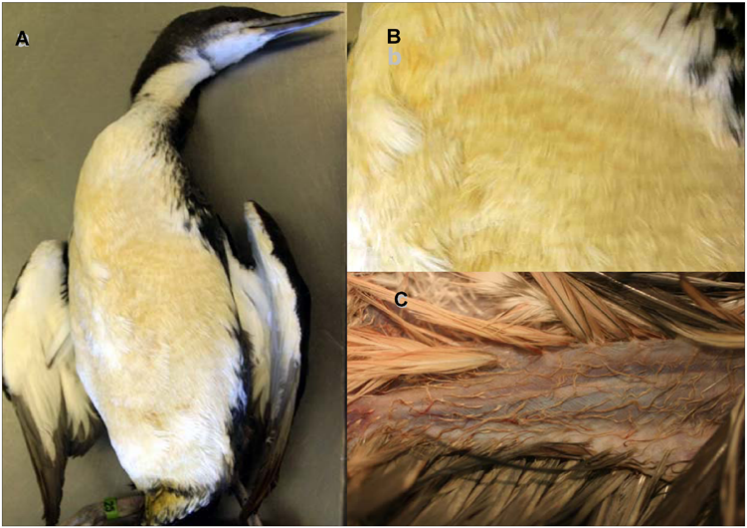
Dead seabirds with signs of protein-induced loss of waterproofing. A) A Pacific loon found dead in Monterey Bay, CA in November, 2007 with yellow-green staining of ventral breast feathers typical of over 700 marine birds affected by this event. B) Closer view of the stained feathers from (A), illustrating the yellow-green discoloration and the oily appearance of affected feathers. C) Discolored, wet and matted feathers on the ventral wing of a western grebe recovered during the same stranding event.

Live-stranded birds were severely hypothermic, wet and hypoglycaemic, with variable patches of a slimy pale yellow-green material on their feathers (Figure 1) with a “bathtub ring” distribution, primarily on the breast area and ventral surfaces of wings and tail. Freshly stranded birds had a pungent odour similar to that of linseed oil while still wet, but with time, this material dried, leaving a fine, pale yellow crust with minimal smell. Clinical blood values for fulmars (e.g., packed cell volume = 24 to 37) indicated anaemia. External lesions in live birds were minor, other than diffuse pallor (i.e., anaemia) and patchy erythema and superficial excoriation of skin that had been in contact the slimy material and beach sand. With supportive care, these abnormalities resolved spontaneously. Upon rinsing, rehydration, warming, and nutritional supplementation, birds regained substantial body mass within 9 days and a high percentage of affected birds (e.g. 42% of fulmars) recovered and were released, suggesting that the slimy substance was minimally toxic, or nontoxic. Postmortem examinations were conducted on 283 birds from the event, including animals that were found dead and those that died during rehabilitation. The majority of necropsied birds were in fair or poor nutritional condition, with little or no subcutaneous fat (91%) and moderate to severe atrophy of pectoralis muscle (85%, n = 220). There were no additional lesions shared by the majority of affected birds at gross necropsy, other than the pallor and skin irritation noted above for the live-stranded birds. No clinical, gross, microscopic or bacteriological evidence for botulism, avian influenza or other infectious or toxic process was detected in birds from this mass-stranding event.

Microscopic examination of tissues from 18 individuals of six species (grebes, fulmars, loons and scoters) revealed few lesions that were common to the majority of affected birds. However, approximately half of the birds had gross and/or microscopic evidence of acute haemorrhage into the pulmonary parabronchi and adjacent pulmonary parenchyma was sometimes pale and hypoperfused. In two affected fulmars, the pulmonary haemorrhage was accompanied by mild, fibrinous airsacculitis. Minimal inflammation was noted in the walls of affected air sacs and no fungal or bacterial infiltration was identified on histopathology or culture, but the respiratory epithelium appeared discontinuous and variably hyperplastic. A few birds had low numbers of tiny (1–3 mm^3^), raised, yellow mucosal plaques in the proximal digestive tract (choana, oropharynx and/or proximal esophagus). Microscopic examination of these lesions revealed nodular infiltrates of pyogranulomatous inflammation containing mixed bacteria and/or small fragments of degenerating nematode parasites. No microscopic evidence of poxviral infection was found. In live birds, these lesions appeared to be self-limiting and resolved while in care.

**Figure 2:**
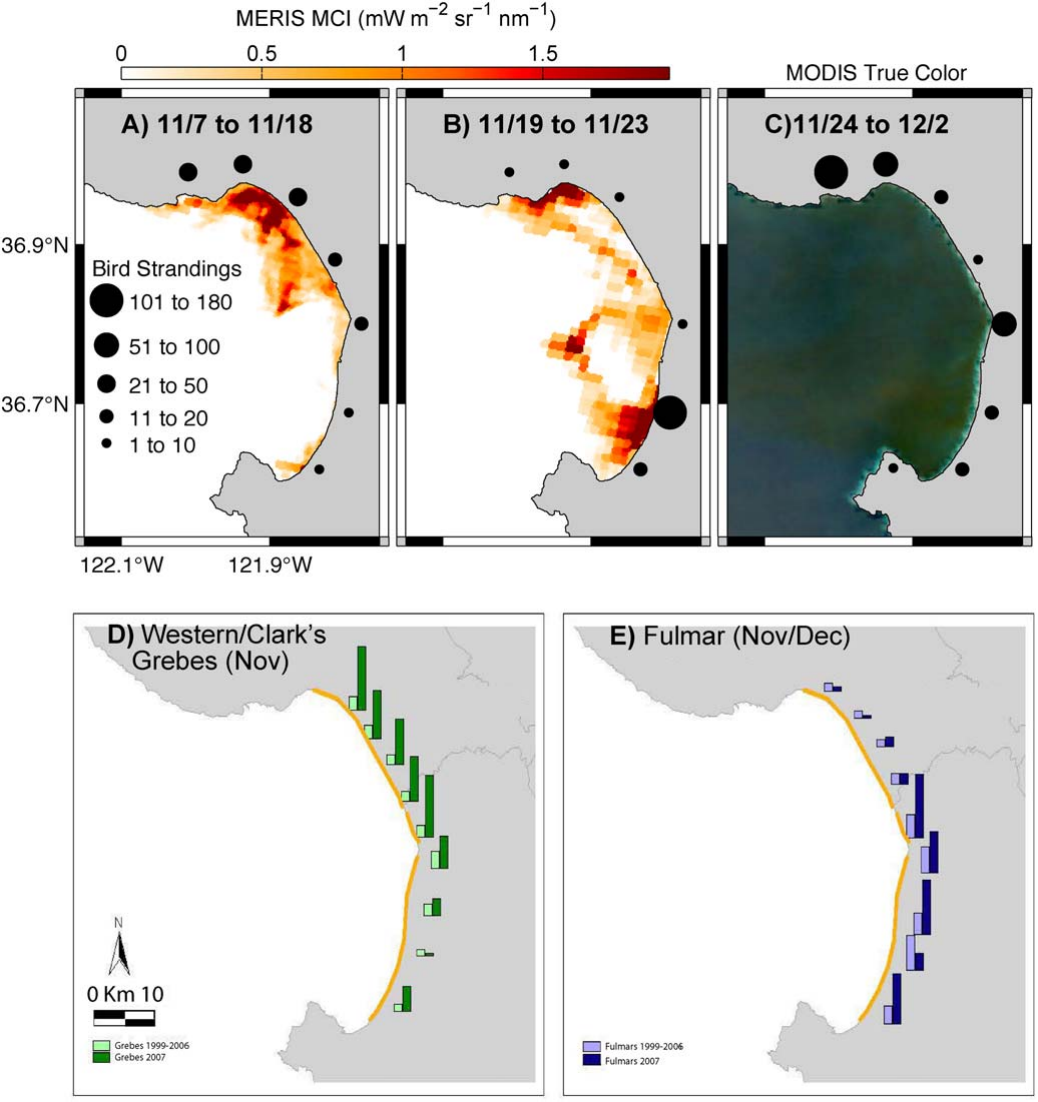
Correspondence between the spatial and temporal patterns of seabird strandings and red tide during November-December 2007 in Monterey Bay. Black circles represent the approximate location and magnitude of morbidity and mortality, and satellite data represent the red tide for panels (A–C). Three pulses of seabird stranding were observed: A) focused along the northern bay, B) focused in the south-central portion of the bay, and C) in both northern and central portions of the bay. In (A) and (B) the MERIS data indicate extreme bloom conditions above MCI level of ∼0.3. Although ideal for red tide detection, MERIS coverage was not adequate for the third pulse. In (C) the reddish discoloration on the MODIS true color image during the third pulse (28 Nov) shows the spatial extent and location of the red tide. Panels (D) and (E) show increased seabird stranding during the November–December 2007 red tide event. Light bars are 10-year average (1997–2006) standardized monthly stranding counts on nine Monterey Bay beaches. Dark bars are 2007 counts for western/Clark's grebes and Northern fulmar, the species most affected during the mass-stranding event. Panel (D) corresponds to the first stranding event, while panel (E) corresponds to the second and third waves of strandings.

Three distinct pulses of live and dead bird strandings occurred in spatial and temporal coincidence with the development and circulation of an intense “red tide”. The first pulse of red tide and bird strandings was most concentrated in northern Monterey Bay (Figure 2A). The second pulse was marked by expansion of the red tide and increased seabird strandings along the southern portion of bay (Figures 2B and 2E). The third pulse occurred in both areas simultaneously (Figure 2C). Standardized periodic Beach COMBER surveys documented marine bird stranding rates 2 to 24 times greater than the 10-year average for Western/Clark's grebes on most northern beaches in Monterey Bay (Figure 2D). These species typically forage along the shoreline where dense patches of senescing algae and foam were observed. In contrast, northern fulmar is a more pelagic species that forages near the center of Monterey Bay. Fulmars appeared to have encountered the red tide and its attendant foam further offshore in the southern and central bay (Figure 2B). Fulmars stranded in numbers 2 to 4 times greater than the 10-year average for the months of November–December, but were more highly localized to specific beaches in southern Monterey Bay (Figures 2E).

**Figure 3:**
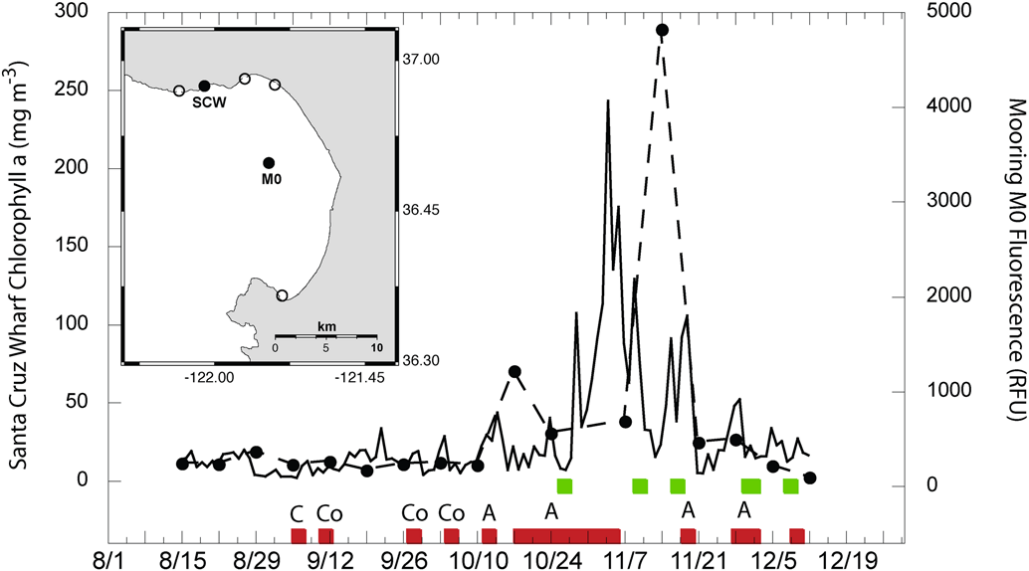
Observations of chlorophyll, fluorescence, phytoplankton species composition, and occurrence of foam and red tide patches were available from a series of stations in Monterey Bay. Solid symbols (dashed line) represent total chlorophyll collected weekly from the Santa Cruz Municipal Wharf from August 15 to December 15, 2007 (labelled SCW on the inset map); the solid line represents daily chlorophyll fluorescence data for the same time period from the M0 mooring. Red bars along the x-axis denote visual observations of red tides from stations depicted in the map (inset), with the letters above the red bars denoting dominant species (C = *Ceratium* spp., Co = *Cochlodinium* spp., A = *Akashiwo sanguinea*). Green bars denote visual observation of foam at the Santa Cruz Municipal Wharf. The bird strandings coincided with the largest red tides (note the chlorophyll and fluorescence) dominated exclusively by *A. sanguinea* and co-occuring with foam.

**Figure 4:**
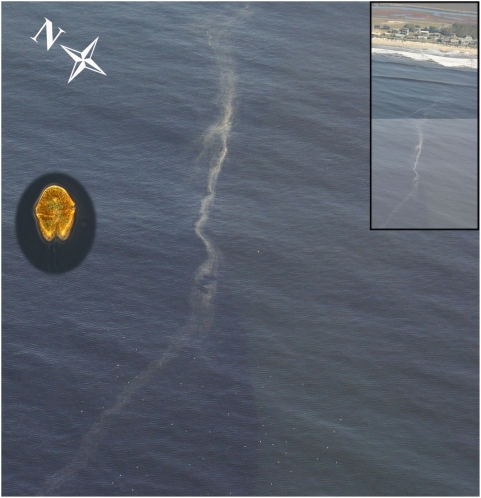
Aerial photograph taken 26 November, 2007 off Pajaro Dunes Colony (rectangular inset) in central Monterey Bay. A dense front of red tide is visible in the left foreground. A distinct line of surface foam is spatially associated with the red tide, along with aggregations of marine birds. Circular inset: a single non-senescent *Akashiwo sanguinea* cell.

**Figure 5:**
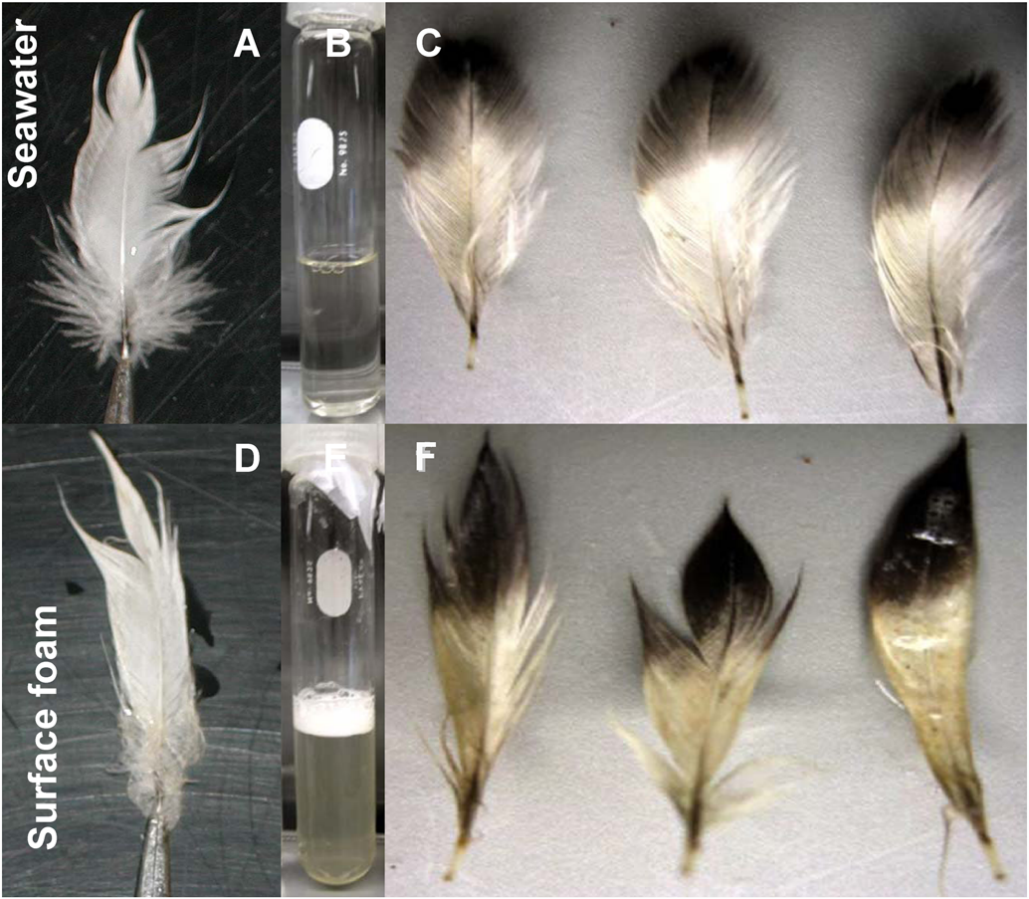
Natural and laboratory produced wetting of normal feathers. A) Normal breast feather from a brown pelican after dipping in clean seawater. Note the minimal wetting and matting of feather barbs. B) Normal seawater after agitation for 30 seconds, then resting for 2 hours. Note the absence of surface foam. C) Normal-appearing breast feathers from a Northern fulmar after dipping in the test tube containing normal seawater. D) Normal breast feather from a brown pelican after dipping in surface foam collected near an *A. sanguinea* bloom in Monterey Bay. Contact with the foam resulted in severe wetting and matting of feather barbs. E) Senescent laboratory culture of *A. sanguinea* after agitation for 30 seconds, then resting for 2 hours. Note the persistence of a thick surface layer of foam. F) Normal breast feathers from a Northern fulmar after dipping in the surface foam derived from the senescent *A. sanguinea* culture, resulting in severe wetting and matting of feather barbs.

Various programs monitor phytoplankton species composition in the Monterey Bay region, including the California Program for Enhanced Regional Monitoring of Phycotoxins (Cal-PReEMPT), which collects weekly samples for species and toxin analysis from the Santa Cruz Municipal Wharf. During autumn 2007, there were a series of dinoflagellate blooms, including several red tides, culminating in the large scale event reported herein (Figure 3). Plankton samples collected at various Monterey Bay nearshore and offshore stations during November 2007 were consistently dominated (both numerically and by biomass) by *Akashiwo sanguinea* ( = *Gymnodinium sanguineum*), an autotrophic dinoflagellate (Figure 4, circular inset). Significantly elevated levels of other biotoxin-producing dinoflagellates were not detected in Monterey Bay during this event. *Akashiwo sanguinea* is capable of producing large quantities of mycosporine-like amino acids (MAAs), which are water-soluble and serve as powerful surfactants [8]. *Akashiwo sanguinea*-associated blooms have also been linked to coral bleaching due to the formation of proteinaceous material [18], similar to that found in this event. In this event surface foam accumulated adjacent to the most densely concentrated portions of the bloom along frontal edges (Figure 4) and in the surf zone where wave action presumably produced it through turbulent mixing of the organic-rich seawater. Affected birds were observed swimming in the thick foam near the shoreline. When normal seabird feathers were dipped in foam collected from near Santa Cruz Wharf on December 5, 2007, the contour feathers and underlying down lost their normal water repellency (Figure 5D) and became soaked. Clean seawater collected from the Santa Cruz Wharf (Figure 5A, 5B, 5C) and deionized (Milli-Q) water did not have this effect.

Extracts of seawater from four areas in northern Monterey Bay heavily impacted by the red tide were analyzed for polar and non-polar compounds by gas- and liquid chromatography-mass spectroscopy and were found to be negative for petroleum compounds, commercial surfactants, pesticides, domoic acid, okadaic acid, and microcystin toxins. However, samples of the co-occurring surface foam present at these same sites contained significant concentrations of an organic compound with a predominant chromatographic peak at 1230 mw, corresponding to a m/z 616 dimer composed of carbon, hydrogen, nitrogen and oxygen. Fragmentation of the m/z 616 parent compound resulted in loss of several m/z 59 fragments characteristic of peptide chains. When foam samples were allowed to sit for several days at ambient temperature, these peptides became difficult to detect. Analysis of the surface foam and feathers from affected birds coated with the yellow-green proteinaceous material revealed high concentrations of the same 616 m/z protein dimer, but seawater collected from unaffected areas and normal feathers of healthy seabirds of the same species were negative for this compound.

Samples of the foam were also analyzed by UV-VIS spectroscopy, and exhibited characteristic UV-absorption spectra indicative of MAAs that are commonly associated with dinoflagellate blooms. As with the proteins detected by mass spectrometry, the MAA-like absorption features degraded (within 24 hours) when stored in the dark at 4°C. Uni-algal, xenic laboratory cultures of *A. sanguinea* isolated from Monterey Bay produced compounds with the same UV-absorption signature (indicative of MAA compounds) as the field-collected algal, feather and foam samples. When senescent laboratory cultures of *A. sanguinea* were vigorously agitated for 30 seconds and then left to rest for 2 hours, they produced surface foam (Figure 5E) that had the same wetting properties on normal bird feathers as the naturally occurring foam (Figure 5F); the foam was still intact at the end of the two hour rest period. In contrast, agitation of healthy *A. sanguinea* cultures did not result in foam production beyond transient bubble formation which did not persist for more than ca. 15 seconds, and exposing normal bird feathers to vigorously shaken healthy cultures or to sterile seawater media did not result in wetting.

## Discussion

Northeastern Monterey Bay has previously been identified as a “red tide incubator” [19]. Analysis of remote sensing data (ocean color, temperature and currents) suggests relatively weak circulation, and presumably enhanced retention of the prolonged red tide bloom during the marine bird stranding event. The bloom was first detected by satellite in this area before the harmful effects on the marine birds were detected, and it persisted throughout the event. The first and third pulses of marine bird live strandings and mortality occurred primarily within this incubator region, and mainly impacted bird species that typically forage close to shore (Figure 2). In contrast, the second pulse of strandings was composed mainly of a more pelagic species (northern fulmars), and the timing and location of these fulmar strandings coincided with the offshore advection of the algal plume.

Because the gross lesions in affected birds were subtle and nonspecific, and the worldwide distribution of *A. sanguinea* is broad, prior stranding events resulting from these blooms may have previously gone unrecognized. Both loons and grebes undergo molt shortly after arriving in Monterey Bay. As a result, many birds were unable to fly effectively and thus could not adequately compensate for loss of water repellency and body heat. Birds may have also been nutritionally stressed due to lower winter food availability, which may have exacerbated hypothermia once their plumage was fouled by the HAB residue. This hypothesis is supported by the high proportion of emaciated, fresh dead birds that were recovered during the event. Live-stranded birds may have simply been in better nutritional condition prior to fouling.

The proteinaceous material was relatively easy to remove from the birds using simplified oil spill washing procedures and the recovery rate for live-stranded animals was high, compared to typical survival rates for similar species with external fouling by petroleum compounds. Feather fouling, reduced mobility, and hypothermia appear to have acted as cumulative stressors for the more debilitated birds.

In addition, some birds showed gross or microscopic evidence of acute haemorrhage into the lungs and patchy fibrin deposition in air sacs that could be consistent with oxidative damage to respiratory epithelium. A similar, but more severe pulmonary lesion is reported in birds exposed to aerosolized products from overheated polytetrafluorethylene (Teflon)-coated pans [20]. Although the pathogenesis could not be identified in this case, one important possibility that merits investigation is transient exposure to an aerosolized component of the surface slime. Of note, some animal care personnel also reported mild respiratory irritation after contact with heavily soiled birds during this event. Aerosolization of toxins during bloom events have been reported for other biotoxins, including brevetoxins [21] and microcystins [22], and aerosolization of peroxides has been reported for *Heterosigma akashiwo* [23], which is also produced by *A. sanguinea* [24]. If a respiratory toxicant, such as peroxide, is found to be liberated from the sticky material resulting from these senescing blooms, it may have important implications for husbandry of heavily soiled birds in enclosed spaces to optimize avian and human health. Because captive seabirds are highly susceptible to ubiquitous and opportunistic respiratory pathogens like the fungus *Aspergillus fumigatus*, any factors that could potentiate these infections should be avoided.

Mass stranding of recently molted grebes, loons and scoters with wet, “apparently oiled” feathers also occurred in Monterey Bay in 1997 and was temporally associated with an uncharacterized red tide. Red tides of similar magnitude were observed in Monterey Bay in August-September 2004 [25] and in September 2006, with no substantial increase in reports of bird mortalities. The lateness, duration and intensity of the 2007 *A. sanguinea* red tide coupled with increased nearshore abundance of wintering birds evidently accentuated the impacts and detection of the unusual effects of this algal bloom.

Prediction of the harmful effects of harmful algal blooms requires a better understanding of linkages between climate variability, phytoplankton blooms and natural history of higher animals. An “unprecedented” red tide produced by *A. sanguinea* in San Francisco Bay during September 2004 was attributed to an upper-atmosphere high-pressure anomaly off the U.S. west coast, following a summer of weak coastal upwelling [26]. The circulatory, nutrient, weather and surf conditions that existed in November 2007 favoured an especially intense and prolonged event that produced large amounts of foam from a very large, senescing dinoflagellate bloom by extending the red tide into a period of increased winter swell. It remains to be seen whether the combination of events we describe for 2007 is a result of climate change, but the frequency, amplitude and duration of “red tides” have increased substantially within Monterey Bay since 2004 [27] and continue to increase globally [5], suggesting that the harmful effects of dinoflagellate blooms may become more common.

## Materials and Methods

### Bird stranding data

Live and dead bird stranding data were spatially binned according to where the stranding locations formed clusters, and were temporally binned according to three distinct pulses of stranding activity. The two most affected species (western/Clark's grebes and northern fulmars) were examined in more detail. The Beach Coastal Ocean Mammal and Bird Education and Research Surveys program [28] record dead strandings during monthly beach surveys. New deposition (i.e., new birds km^−1^) from November and December 2007 surveys are presented in relation to the monthly mean (1997–2006) stranding data from the same beaches for the same months.

### Pathology

A total of 283 stranded marine birds of fourteen species from this incident received gross postmortem examinations, with assessment of sex, age nutritional condition and major gross lesions. Species composition of necropsied birds included 174 Northern fulmars (*Fulmarus glacialis*), 35 Western grebes (*Aechmophorus occidentalis*), 24 surf scoters (*Melanitta perspicillata*), 12 common murres (*Uria aalge*), 9 Brandt's cormorants (*Phalacrocorax penicillatus*), 9 Clark's grebes (*A. clarkii*), 5 Pacific loons (*Gavia pacifica*), 4 double-crested cormorants (*P. auritus*), 2 sooty shearwaters (*Puffinus griseus*), 2 Western gulls (*Larus occidentalis*), 2 rhinoceros auklets (*Cerorhinca monocerata*), 1 brown pelican (*Pelecanus occidentalis*), 1 California gull (*L. californianus*), 1 common loon (*G immer*), 1 red-throated loon (*G. stellata*), and 1 forked-tailed storm-petrel (*Oceanodroma furcata*). The majority of dead birds had been frozen and were examined after the event ended. Full necropsy, including bacterial culture and microscopic examination of paraffin-embedded, H&E-stained tissues from all major organs was performed for 18 birds, including grebes, loons, scoters and fulmars. Special stains for bacteria and fungi were also performed for selected cases in order to better characterize microscopic lesions. Finally, soiled feathers were collected from a subset of the most heavily affected birds to facilitate chemical analysis.

### Description of the red tide

We primarily used 300 m and 1200 m images from the MERIS satellite sensor. Average MCI [29], [30] corresponding to the three pulses of bird strandings was calculated. For the first stranding period (7–18 November 2007), two images preceding (2–3 Nov) and one image during this pulse (12 Nov) were averaged. Three MCI images (19, 21, 22 Nov) within the second period (19–23 Nov) were averaged. For the third period (24 Nov–2 Dec), MCI coverage was not adequate, and we used 500-m resolution MODIS true-color images.

### Chemical analysis

Foam collected by boat and from shore and material from live and dead stranded birds was analyzed following standard protocols for identification of marine toxins, contaminants, and petroleum products. This included analysis by gas chromatography with ion trap detector (ITD), electron capture detector (ECD), and flame photometric detector (FPD), as well as liquid chromatography/mass spectrometry (LC/MS), LC/MS/MS, and UV/VIS absorption.

### Foam reproduction

A monospecific strain of *Akashiwo sanguinea* (AkaMB1106) was grown on L1 media. The culture was allowed to enter stationary phase and senesce. 500 mL each from senescent and healthy cultures was agitated for approximately 30–60 seconds, until foam was produced in the senescent culture. L1 media with no cells was used as a control.
